# MiR-145-5p arrests the cell cycle by modulating SMAD5/cyclin D1 to inhibit gastric cancer progression

**DOI:** 10.3389/fcell.2025.1619359

**Published:** 2025-08-07

**Authors:** Li Wang, Jianghong Guo, Caixia Lv, Luke Kong, Jing Cui, Zhongshuai Wang, Yuanyuan Guo, Ruirui Jia, Tao Guan, Baofeng Yu, Feng Li

**Affiliations:** ^1^Department of Biochemistry and Molecular Biology, Shanxi Key Laboratory of Birth Effects and Cell Regeneration, MOE Key Laboratory of Coal Environmental Pathogenicity and Prevention, MOE Key Laboratory of Cellular Physiology, Shanxi Medical University, Taiyuan, China; ^2^Department of Central Laboratory, Shanxi Province Cancer Hospital/Shanxi Hospital Affiliated to Cancer Hospital, Chinese Academy of Medical Sciences/Cancer Hospital Affiliated to Shanxi Medical University, Taiyuan, Shanxi, China; ^3^Department of Pathology, Shanxi Province Cancer Hospital/Shanxi Hospital Affiliated to Cancer Hospital, Chinese Academy of Medical Sciences/Cancer Hospital Affiliated to Shanxi Medical University, Taiyuan, Shanxi, China; ^4^Department of Gastroenterology, Shanxi Province Cancer Hospital/Shanxi Hospital Affiliated to Cancer Hospital, Chinese Academy of Medical Sciences/Cancer Hospital Affiliated to Shanxi Medical University, Taiyuan, China; ^5^Basic Medical College, Shanxi Medical University, Taiyuan, Shanxi, China; ^6^Department of Medical Laboratory, Jincheng People’s Hospital, Jincheng, China; ^7^Department of Cell Biology, The Province and Ministry Co-sponsored Collaborative Innovation Center for Medical Epigenetics, School of Basic Medical Sciences, Tianjin Medical University, Tianjin, China; ^8^Renal Department Blood Purification Center, Shanxi Provincial People’s Hospital, Taiyuan, China; ^9^Department of Hematology, Shanxi Hospital Affiliated to Cancer Hospital/Shanxi Province Cancer Hospital, Cancer Hospital Affiliated to Shanxi Medical University/Chinese Academy of Medical Sciences, Taiyuan, Shanxi, China; ^10^Shanxi Provincial Key Laboratory of Lymphoma Precision Diagnosis and Treatment Research, Taiyuan, Shanxi, China

**Keywords:** miR-145-5p, Smad5, gastric cancer, cyclin D1, cell cycle

## Abstract

**Purpose:**

This study aimed to explore the dysregulation of miR-145-5p in gastric cancer (GC) and its effects on the proliferation and cell cycle of GC cells, exploring the potential regulatory mechanism of miR-145-5p in GC.

**Methods:**

In this study, the TCGA database combined with Microarray was used to detect differentially expressed microRNA (miRNA) in GC tissues and cells. Quantitative real-time (qRT)-PCR was used to further verify the expression of miR-145-5p in GC cells and the 41 pairs of GC tissues and adjacent tissues. A retrospective analysis was conducted on the correlation between miR-145-5p and the clinicopathological characteristics of patients with GC. The proliferation ability and cell cycle of AGS and MKN28 were detected by CCK-8, Edu and flow cytometry. The downstream target genes of miR-145-5p were screened by bioinformatics and further verified by the dual-luciferase reporter assay. Immunohistochemistry was used to detect the expression of SMAD5 in GC tissues. Western blot was used to detect cell cycle-related proteins that were regulated by siRNA SMAD5.

**Results:**

The expression of miR-145-5p was lower in GC tissues and cells compared with adjacent tissues and GES-1, and was related to the poor prognosis of patients with GC. Overexpression of miR-145-5p inhibited the proliferation of GC cells and blocked the cell cycle from G1 phase to S phase. MiR-145-5p targeted SMAD5 to inhibit the proliferation, and arrested the G1/S phase transition of GC cells. Mechanistically, SMAD5 siRNA significantly reduced CCND1 protein expression, Bioinformatics databases predicted that cyclin D1 was the transcription target gene of SMAD5. Moreover, the re-expression of cyclin D1 partially reversed the cell cycle arrest that was induced by SMAD5 depletion in GC cells.

**Conclusion:**

Taken together, these findings reveal a novel role of the miR-145-5p/SMAD5/cyclin D1 axis in modulating cell cycle progression and cell proliferation in GC, which may provide a prognostic biomarker for GC treatment.

## 1 Introduction

Around the globe, gastric cancer (GC) ranks fifth in prevalence and third in deaths from cancer. In 2018, there were more than 1,000,000 new cases of gastric cancer worldwide, and an estimated 783,000 people died from the disease (Bray et al., 2018). The diagnosis, surgical methods, and targeted therapy have all advanced largely in the last few decades. However, the 5-year mortality rate for advanced gastric cancer is still 30%–50% (Hamashima et al., 2015). To achieve precise treatment of gastric cancer, the possible molecular mechanisms that underlie GC occurrence and development of GC are a pressing issue that warrants attention.

As small noncoding RNAs, microRNAs (miRNAs) regulate translation by binding to the 3′-untranslated region (3′UTR) of the target mRNA and even leading to degradation of the target mRNA (Zhao et al., 2023). miRNAs control the initiation, metastasis, angiogenesis, and treatment resistance of cancer pathogenesis. GC prognosis and diagnosis may benefit from using miRNAs as biomarkers. Cao et al. found that poor OS in GC patients is predicted by low expression of the miR194-2HG (Cao et al., 2024). MiR-21 is overexpressed in GC tumour tissue, and higher expression of miR-21 is related to poorer survival in GC patients (Sekar et al., 2016).

MiRNAscope, which depends on tissue microarrays, was used to analyze differentially expressed miRNAs in GC and matched nontumor tissues in order to screen for the key miRNAs involved in GC carcinogenesis. The results showed downregulated miR-145-5p in GC tissues. MiR-145-5p has been reported to be a tumour suppressor in a variety of cancers, including breast cancer, bladder cancer and lung cancer (Dong et al., 2021; Gan et al., 2017; Wang et al., 2019; Zhu et al., 2019). The overexpression of miR-145-5p induces cell apoptosis and prevents proliferation and migration (Zeinali et al., 2020). In addition, lncRNAs and circRNAs bind to miR-145-5p as competitive endogenous RNAs (ceRNAs) and participate in the development and progression of GC (Liu et al., 2019; Ren et al., 2017). All of the above-mentioned findings illustrate the importance of miR-145-5p in GC tumourigenesis. Bai HX et al. reported that miR-145-5p plays an important role in this disease by targeting serine family E member 1 (SERPINE1), regulating the extracellular signal-regulated kinase-1/2 (ERK1/2) pathway and directly affecting GC progression (Bai et al., 2024). Still unknown, though, is the precise function of miR-145-5p and what mechanism underlies it.

In order to better understand how miR-145-5p controls GC proliferation and cell cycle, as well as the feasibility of employing miR-145-5p as a biomarker for GC diagnosis as well as prognosis, we conducted this study.

## 2 Materials and methods

### 2.1 Clinical samples

Between 2018 and 2019, 41 people who had radical GC resections at the Cancer Hospital Affiliated with Shanxi Medical University’s Department of Gastric Surgery provided the primary GC samples used in this investigation. Prior to surgery, adjuvant chemotherapy was not administered to the patients. All participants signed consent forms, and the study was approved by the Cancer Hospital’s Ethics Committee, which is affiliated with Shanxi Medical University.

### 2.2 Cell culture and cell transfection

The Chinese Academy of Sciences’ Cell Bank provided all of the GC cell lines (MKN28, HGC-27, MKN45, and AGS) as well as the GES-1 cell line (human gastric epithelial cells). Ten percent foetal bovine serum (Wisent, Canada), along with antibiotics (1% streptomycin/penicillin; Gibico), were added to RPMI-1640 (Gibco, United States) at 37°C with 5% CO_2_.

Sangon Biotech (Shanghai, China) synthesized inhibitors and mimics of miR-145-5p. As directed by the manufacturer, Lipofectamine 2000 (Invitrogen) was utilized for transfection. GenePharma (Shanghai, China) synthesized small interfering RNAs (siRNAs) that target SMAD5. CCND1-overexpressing cells were generated via the pcDNA 3.1 plasmid (abmgood, China). The sequences of the transfection reagents are shown (Supplementary Table S1).

### 2.3 Quantitative real-time polymerase chain reaction

We used TRIzol reagent (Invitrogen, Thermo Fisher Scientific, United States) to extract RNA from GC and paracancerous tissues as well as cell lines. We used PrimeScript RT reagents (Takara, Otsu, Japan) to reverse transcribe the RNA into cDNA, and a LightCycler 480 II Real-Time PCR System was used to perform quantitative real-time polymerase chain reaction (qRT-PCR) using SYBR Green reagents (Takara, Otsu, Japan). The 2^−ΔΔCT^ method determined relative miR-145-5p expression, which was normalized to that of U6.The 2^−ΔΔCT^ method determined relative Gene expression, which was normalized to that of GAPDH. Every sequence that was used is displayed in Supplementary Table S2.

### 2.4 Western blotting

Using RIPA buffer enhanced with 1% phosphatase and protease inhibitors, we extracted protein from cells (Beyotime, Jiangsu, China). Using a BCA protein assay kit (Beyotime, Jiangsu, China), we calculated the protein concentration. Polyvinylidene fluoride (PVDF) membranes held the protein after separation on 10% SDS-PAGE gels. Following 2 h of blocking with 5% nonfat milk in TBST, we incubated the membrane with primary antibody at 4°C for the entire night and with HRP-conjugated secondary antibodies at room temperature for 2 h. Using an enhanced chemiluminescence (ECL) kit (ABBkine, Wuhan, China), we identified the membrane following three TBST washes.

The primary antibodies were: SMAD5 (Proteintech, 67052-1-Ig, 1:2,000), cyclin D1 (Proteintech, 60186-1-Ig, 1:10,000), CDK4 (BBI, D120396, 1:500), CDK6 (BBI, D120398, 1:500), cyclin E1 (Proteintech, 11554-1-AP, 1:1,000), and P21 (Bioss, bs-55160R, 1:500).

### 2.5 5-ethynyl-2′-deoxyuridine incorporation and cell counting kit-8 (CCK-8) assays

In 96-well plates, we seeded GC cells at 2,000 cells/well for the CCK-8 assay. Following 2 h of incubation at 37°C, CCK-8 (DOJINDO, Tokyo, Japan) was added to the cells after they had had time to attach. Then, we measured the absorbance at 450 nm in a microplate reader (Spectra Rainbow, Tecan). The cells were photographed using a fluorescence microscope, and the EdU incorporation assay was carried out using an EdU kit (RiboBio, Guangzhou, China).

### 2.6 Cell cycle analysis

Using a Cell Cycle Detection Kit (Solarbio, China), we identified the cell cycle distribution through flow cytometry. Six-well plates were used to cultivate the GC cells, which were harvested at about 80% confluency. The cells underwent a 12-h fixation in 70% cold ethanol, a 100 μL of RNase A solution precipitation, a 30-min suspension in a water bath at 37°C, propidium iodide (PI) staining, and flow cytometry (BD Biosciences, United States).

### 2.7 Immunohistochemical staining

Each sample was sectioned into 5 μm pieces after being embedded in paraffin and fixed in 4% formalin. Primary antibodies (SMAD5, CCND1, Proteintech 67052-1-Ig, and 60186-1-Ig) were incubated on the sections for an entire night at 4°C. The sections were then incubated for 30 min at room temperature with secondary antibodies. Following that, the sections were stained for 5 min using DAB solution. The percentage of positive cells in the IHC staining was used to assign a score: none = 0, <25% = 1, 26%–50% = 2, 51%–75% = 3, and >75% = 4. Cell staining intensity was analyzed as follows: strong = 3, moderate = 2, weak = 1, and negative = 0. To get the total score range of 0–12, we multiplied the percentage score by the staining intensity score.

### 2.8 Luciferase reporter assay

We used the pmirGLO vector to construct the SMAD5 3′UTRs (wild and mutant types) and TargetScan to predict the binding sequences of SMAD5 3′UTRs and the miR-145-5p. We introduced two reporter genes into 293T cells, and 24 h later, we introduced miR-145-5p NC or miR-145-5p mimics. The fluorescence intensity was detected 48 h later.

### 2.9 Xenograft tumour model

Firstly, plasmid vectors containing the target miRNA sequence (miR-145-5p mimics and miR-145-5p NC) and purinomycin were constructed. They were transfected into AGS cells through liposome transfection. Antibiotics were added 48 h later for screening for 7–14 days. After obtaining the stably transfected cells, the expression of miRNA were verified by qPCR.

The Shanxi Medical University-affiliated Cancer Hospital’s Committee of Animal Ethics gave its approval to the animal experimentation protocols. We bought 5-week-old female BALB/c nude mice from Beijing Vitonlihua Experimental Animal Technology Co., Ltd. (Beijing, China). After being prepared at 1 × 10^7^ cells/100 μL, AGS cells that were stable overexpressing the miR145-5p or miR145-5p NC mimics were subcutaneously injected into the mice’s right posterior flank. Every 5 days, we measured tumour volumes and assessed using the volume = (length × width^2^)/2 formula.

### 2.10 Statistical analysis

We carried out statistical analyses using either GraphPad Prism 8 or SPSS 20.0. ANOVA and Student’s t tests compared the mean values. We examined associations of SMAD5 expression and cyclin D1 with miR-145-5p expression usingg Pearson’s correlation analysis. T tests was used to examined associations of clinicopathological features with miR-145-5p expression. Using log-rank tests and the Kaplan-Meier method, survival was computed. Three separate experiments’ means ± SDs are shown in the bar graph. It was considered statistically significant when the *P*-value was less than 0.05 (*, *P* < 0.05; **, *P* ≤ 0.01; and ***, *P* ≤ 0.001).

## 3 Results

### 3.1 Downregulated miR-145-5p in cell lines and GC tissues, and low miR-145-5p predicts poor prognosis

**FIGURE 1:**
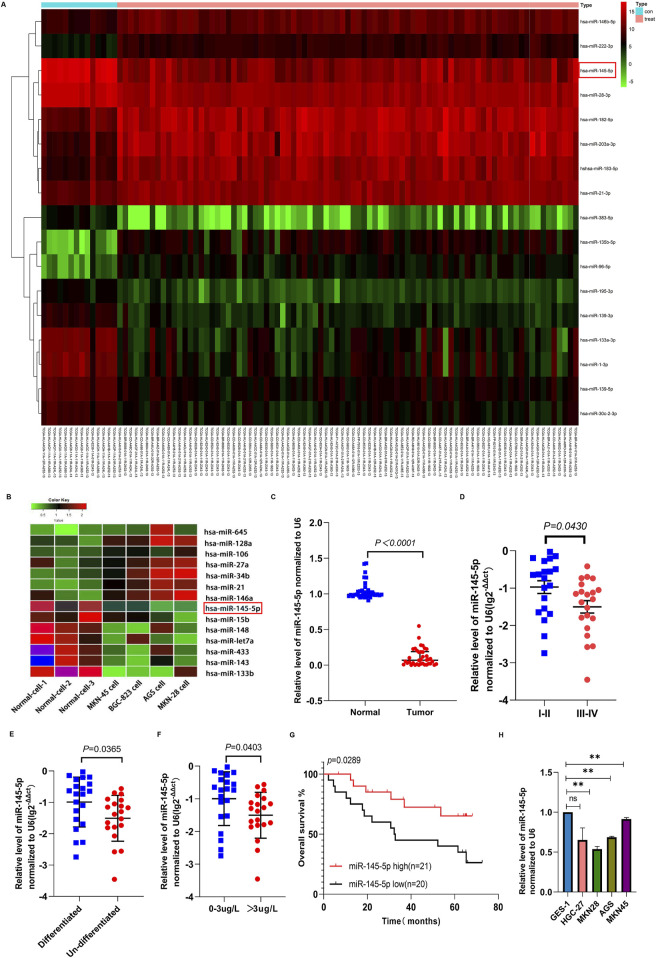
The expression of miR-145-5p in gastric cancer tissues and cell lines and its correlation with clinical characteristics. **(A)** Heatmap of miRNAs differentially expressed between normal and GC cancer samples in the TCGA database. **(B)** Heatmap of the miRNAs differentially expressed in normal gastric epithelial cells (GES-1) and GC cell lines using miRNA array. Red represents upregulation and green represents downregulation. **(C)** The expression of miR-145-5p was verified in 41 pairs of GC and matched adjacent tissues via qRT-PCR. **(D)** The association between miR-145-5p expression and TNM stage. **(E)** Association between miR-145-5p expression and histological type. **(F)** Association between miR-145-5p expression and CEA levels. **(G)** Kaplan-Meier plots for differential expression of miR-145-5p; the median value was used as the cut-off value. **(H)** Expression of miRNA-145-5p in GC cell lines, including AGS, MKN-28, HGC-27, and MKN-45, as well as the (GES-1). The graph represents the mean ± SD; *p < 0.05, **p < 0.01, and ***p < 0.001 versus the control.

Using the TCGA database, we examined the miRNA expression in 14 normal tissues and 85 GC tissues. Figure 1A shows that the expression of 17 miRNAs varied between the samples (Figure 1A). Next, we used the miRNA microarray assay to screen for differentially expressed miRNAs in four GC cell lines as well as normal gastric epithelial cells (GES-1). Seven miRNAs were downregulated in GC cell lines. When contrasted with the GES (Biomarker Technologies, Beijing, China), miR-145-5p was ranked highest (Figure 1B).

In order to corroborate the microarray analysis findings, we used qRT-PCR analysis to confirm that 41 cancer tissues expressed miR-145-5p in comparison to nearby nontumor tissues. GC tissues showed lower relative miR-145-5p expression than nearby nontumour tissues (P < 0.001) (Figure 1C). Pathological stage, CEA level, and histological type were all strongly linked to low miR-145-5p expression, according to our analysis of clinicopathological features (P < 0.05) but not to sex, age, lymph node metastasis, tumour size, or blood vessel invasion (Table 1, P > 0.05). Stage III–IV GC tissues showed significantly less miR-145-5p expression than stage I–II GC tissues (P = 0.043) (Figure 1D). Differentiated tumours expressed more miR-145-5p than did undifferentiated tumours (P = 0.0365) (Figure 1E). miR-145-5p expression was also lower in people having raised CEA levels (>3 μg/L) than in people having normal levels (P = 0.0403) (Figure 1F). People having with lower miR-145-5p levels showed a lower overall survival rate than those having higher levels (P = 0.0289), according to Kaplan–Meier survival analysis (Figure 1G). We then found that many GC cell lines expressed miR-145-5p. Compared with GES-1, AGS, HGC27, MKN28, and MKN45 cell lines showed lower miR-145-5p levels (Figure 1H), suggesting that miR-145-5p might prevent GC progression. When combined, these findings imply that reduced miR-145-5p expression is linked to more advanced GC progression and may be utilized as a prognostic indicator for GC patients.

**TABLE 1:** Correlation between miR-145-5p expression and the clinicopathologic parameter of 41 GC patients.

Clinicopathologic	n = 41	miR-145-5p		p value
Parameter		Lower (n = 2)	Higher (n = 2)	
Age (years)				0.272
≥60		12 (29.27%)	9 (21.95%)	
<60		8 (19.51%)	12 (29.27%)	
Gender				0.413
Male		18 (43.90%)	17 (41.46%)	
Female		2 (4.88%)	4 (9.76)	
CEA level				0.007*
0–3 μg/L		7 (17.07%)	15 (36.59%)	
>3 μg/L		14 (34.15%)	5 (12.20%)	
Histological type				0.019*
Differentiated		7 (17.07%)	14 (34.15%)	
Un-differentiated		14 (34.15%)	6 (14.63%)	
Pathological stage				0.043*
I + II		7 (17.07%)	13 (31.71%)	
Ⅲ + Ⅳ		14 (34.15%)	7 (17.07%)	
Lymphnode metastasis				0.929
N0		5 (12.20%)	5 (12.20%)	
N1–N3		16 (39.02%)	15 (36.59%)	
Tumor size (cm)				0.675
<3.5		2 (4.88%)	2 (4.88%)	
≥3.5		19 (46.34%)	18 (43.90%)	
Blood vessel invasion				0.058
Negative		13 (31.71%)	7 (17.07%)	
Positive		7 (17.07%)	13 (31.71%)	

*p < 0.05.

### 3.2 MiR-145-5p suppresses tumour growth *in vivo* as well as GC cell proliferation *in vitro*

**FIGURE 2:**
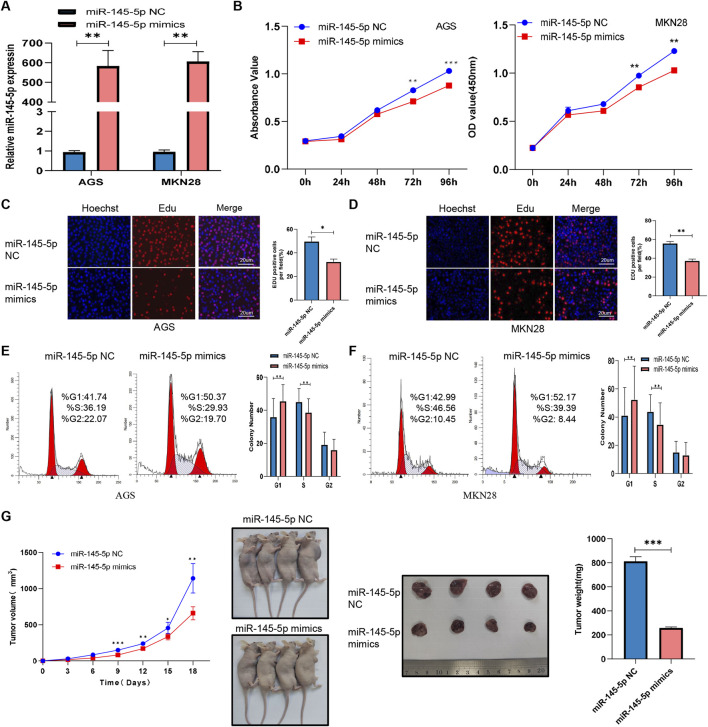
MiR-145-5p suppresses GC cell proliferation *in vitro* and *in vivo*. **(A)** Expression of miR-145-5p in AGS and MKN28 cells after transfection with miR-145-5p mimics. **(B)** A CCK-8 assay revealed that the overexpression of miR-145-5p repressed GC cell proliferation in AGS and MKN28 cells. **(C, D)** The EdU assay showing that the overexpression of miR-145-5p repressed GC cell proliferation in AGS and MKN28 cells. **(E, F)** Flow cytometry assay showing that the overexpression of miR-145-5p arrested the cell cycle in the G1 phase. **(G)** Xenograft tumours were generated in nude mice, and the weights and volumes of the subcutaneous tumours were measured every 3 days. The graph represents the mean ± SD; *p < 0.05, **p < 0.01, and ***p < 0.001 versus the control.

Once miR-145-5p expression in GC was verified, we looked into how it affected the biological functions in GC cells. MKN28 and AGS cell proliferation was markedly suppressed by transfection of miR-145-5p mimics, according to CCK-8 assays (Figures 2A, B). During the S phase, we integrated the thymidine analog EdU into chromosomal DNA replication. Additionally, EdU incorporation assays showed that miR-145-5p mimics slowed down the rate at which EdU was integrated in GC cells (Figures 2C, D). We then used flow cytometry to evaluate the cell cycle distribution in order to ascertain whether the miR-145-5p mimics disrupted the cell cycle. In the AGS as well as MKN28 cell lines, the miR-145-5p mimics clearly raised the proportion of G1/G0 phase cells and lowered the proportion of S phase cells (Figures 2E, F). To find out how miR-145-5p affected GC cell growth *in vivo*, we carried out a xenograft mouse assay. Nude mice received subcutaneous injections of AGS cells that had been stably transfected with either miR-145-5p NC (n = 5) or miR-145-5p mimics (n = 4). Xenograft tumours that were generated from the miR-145-5p mimic AGS cells exhibited slower growth, with a smaller tumour volume and lighter weight being observed compared to tumours generated from the miR-145-5p NC (Figure 2G).

### 3.3 SMAD5 is a downstream miR-145-5p target

**FIGURE 3:**
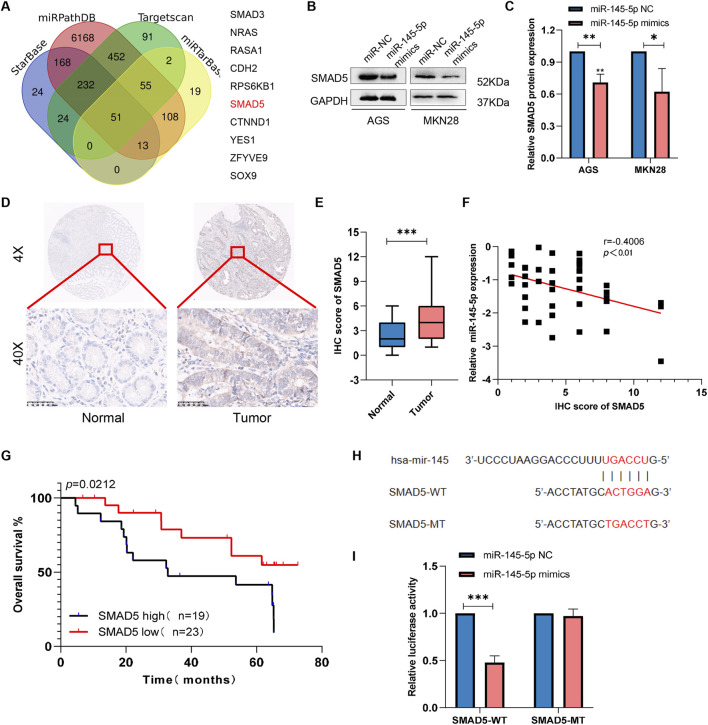
SMAD5 is a downstream target of miR-145-5p. **(A)** Venn diagram showing 10 downstream target genes of miR-145-5p that were identified via TargetScan, miRDB, miRWalk, and miRTarBase. **(B, C)** The expression of SMAD5 in miR-145-5p-overexpressing GC cells. **(D)**. Representative images of immunohistochemical (IHC) staining of SMAD5 in gastric cancer and adjacent paracancerous tissues. **(E)** Total IHC scores of SMAD5 in human gastric cancer and adjacent paracancerous tissues. **(F)** The correlation between miR-145-5p and SMAD5. **(G)** OS analysis based on SMAD5 expression in 41 GC patients; the median SMAD5 expression level was used as a cut-off. **(H)** Predicted binding sites of miR-145-5p to SMAD5. **(I)** miR-145-5p and SMAD5 bind to each other via the predicted region according to a dual luciferase reporter assay. The graph represents the mean ± SD; *p < 0.05, **p < 0.01, and ***p < 0.001 versus the control.

Using four databases, including miRPathDB (https://mpd.bioinf.uni-sb.de/overview.html), TargetScan (https://www.targetscan.org/vert_80/), starBase (https://rnasysu.com/encori/), and miRTarBase (https://mirtarbase.cuhk.edu.cn/∼miRTarBase/miRTarBase_2022/php/index.php), we discovered that SMAD5 is a target gene candidate controlled by miR-145-5p (Figure 3A). In order to ascertain whether SMAD5 expression is connected to miR-145-5p, we carried out Western blotting. We discovered low SMAD5 expression in the cell lines that overexpressed miR-145 than in the miR-145-5p NC cells (Figures 3B, C). To investigate the clinical significance of SMAD5 expression in GC patients, immunohistochemical (IHC) staining was used to detect SMAD5 expression in a human tissue array of the abovementioned 41 clinical gastric cancer tissues and adjacent nontumour tissue samples. Blinded to the clinical data, two pathologists independently scored the tissues’ IHC staining using the semiquantitative H score, taking into account both the percentage of positive cells and the color reaction’s intensity. Compared to nearby nontumor tissues, we discovered that GC tissues had substantially higher levels of SMAD5 expression (Figures 3D, E). In addition, SMAD5 and miR-145-5p were negatively linked (Figure 3F). More importantly, a poor prognosis for GC patients was connected with high SMAD5 expression, according to Kaplan-Meier analysis (Figure 3G). Next, using either the 3′UTR of SMAD5 or a point mutant 3′UTR with predicted miR-145-5p binding sites, we created a pmirGLO luciferase reporter (Figure 3H). MiR-145-5p mimics reduced wild-type SMAD5 luciferase reporter activity but did not affect mutant SMAD5 (Figure 3I). Hence, miR-145-5p targets SMAD5 downstream.

### 3.4 Knockdown of SMAD5 expression inhibits GC cell proliferation as well as cell cycle progression

**FIGURE 4:**
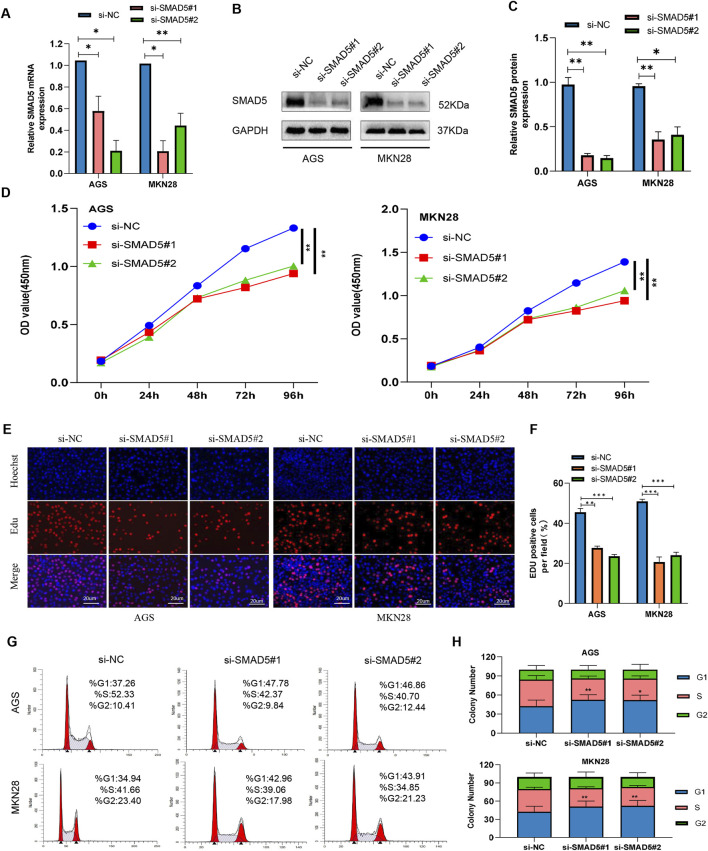
Knockdown of SMAD5 inhibits GC cell proliferation and arrests the G1/S transition. **(A)** SMAD5 mRNA levels in GC cells transfected with negative control siRNA (siNC) or SMAD5 siRNA (siSMAD5). **(B, C)** SMAD5 protein expression in GC cells transfected with siNC or siSMAD5. **(D)** CCK-8 assay showing cell proliferation in transfected GC cells. **(E, F)** EdU assay showing cell proliferation in transfected GC cells. **(G, H)** Flow cytometry revealed that downregulation of SMAD5 arrested the cell cycle in the G1 phase. The graph represents the mean ± SD; *p < 0.05, **p < 0.01, and ***p < 0.001 versus the control.

We created two small inference RNAs (siRNAs) that target SMAD5 and transfected them into GC cells in order to examine the effect of SMAD5 on biological processes within cells. We carried out Western blotting and qRT-PCR to confirm the effectiveness of SMAD5 knockdown in the transfected cells (Figures 4A–C). SMAD5’s function in GC cell proliferation was investigated using the CCK-8 and EdU assays. SMAD5 knockdown significantly suppressed GC cell proliferation (Figures 4D–F). After that, we employed flow cytometry to identify the cell cycle distribution and discovered that GC cell cycle arrest at the G1 phase was caused by SMAD5 knockdown (Figures 4G, H).

### 3.5 MiR-145-5p suppresses GC cell proliferation as well as cell cycle progression via SMAD5

**FIGURE 5:**
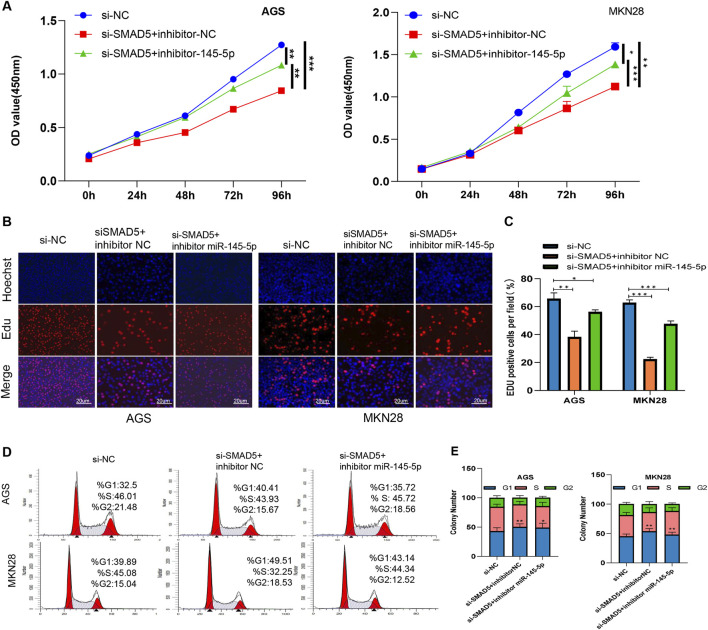
miR-145-5p suppresses cell proliferation and arrests the G1/S phase transition by targeting SMAD5 in GC. **(A)** CCK-8 assay of AGS and MKN28 cell proliferation. **(B, C)** EdU assay revealing cell proliferation in AGS and MKN28 cells. **(D, E)** Cell cycle analysis revealed the cell proliferation ability of AGS and MKN28 cells. *P < 0.05, **P < 0.01 via one-way ANOVA followed by Tukey’s test.

In order to ascertain whether SMAD5 controls miR-145-5p′s effect, we used a combination of si-SMAD5 and the miR-145-5p inhibitor for transfection, followed by the execution of rescue experiments. The EdU and CCK-8 assays showed that the partial reversal of the inhibitory effects on cellular proliferation, resulting from SMAD5 knockdown, was evident upon transfection with the miR-145-5p inhibitor (Figures 5A–C). The G1 phase cell cycle arrest, mediated by SMAD5 siRNA, was partially mitigated by the miR-145-5p inhibitor, as seen in flow cytometry (Figures 5D, E).

### 3.6 SMAD5 regulates cyclin D1’s transcriptional regulation

**FIGURE 6:**
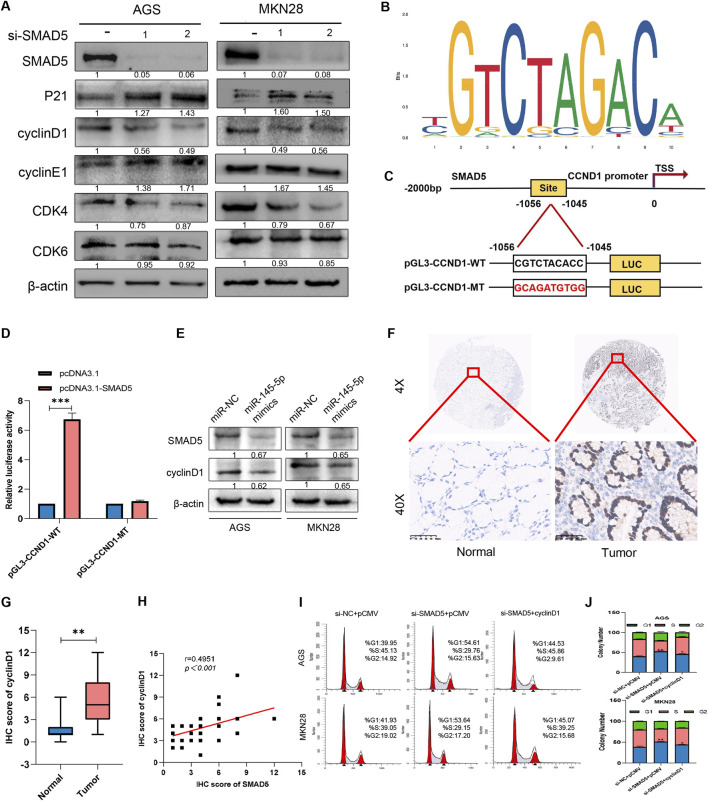
SMAD5 modulates the GC cell cycle via cyclin D1 transcriptional activation. **(A)** Western blot analysis of the expression of cell-related proteins in GC cells transfected with NC siRNA or SMAD5 siRNAs (siSMAD5). **(B, C)** The motif of SMAD5 **(B)**, the presumed binding site (CGTCTACACC) of SMAD5 and the CCND1 promoter (predicted by JASPAR),and its mutation (GCAGATGTGG) **(C)**. **(D)** Luciferase assay of 293T cells co-transfected with CCND1-WT or CCND1-MUT and pcDNA control or pcDNA SMAD5 construct. **(E)** Western blot analysis of the effects of the miR-145-5p mimics on the protein expression of SMAD5 and cyclin D1. **(F)** Representative images of IHC staining of cyclin D1 in GC and adjacent paracancerous tissues. **(G)** Total IHC scores of cyclin D1 in human GC and adjacent paracancerous tissues. **(H)** The correlation between the protein expression of SMAD5 and that of cyclin D1. **(I, J)** Enforced overexpression of cyclin D1 partially reversed the inhibitory effects of siRNA-mediated SMAD5-mediated G1-stage arrest in AGS and MKN28 cells. *P < 0.05, **P < 0.01 via one-way ANOVA followed by Tukey’s test.

After determining SMAD5’s biological function in GC, we explored its potential regulatory mechanisms. When considering that SMAD5 contributes to cell cycle progression, we examined a series of proteins at the G0/G1 checkpoint, including cyclin D1, cyclin E1, CDK4, CDK6 and p21. As shown in Figure 6A, SMAD5 siRNAs significantly reduced cyclin D1 and CDK4 protein levels and increased P21 protein levels; however, no obvious effects were observed on the cyclin E1 and CDK6 proteins.

When considering that CCND1 is a key factor in controlling the G1 phase, we examined whether SMAD5 interacts with cyclin D1. First, we indicated the motif of SMAD5 and predicted the binding site of SMAD5 and CCND1 promoter via JASPAR (Figures 6B, C). When we cloned the fragment of CCND1 promoter containing the putative binding site (CGTCTACACC,-1056 to-1045) into the pGL3 basic vector (CCND1-WT), luciferase activity increased after SMAD5 overexpression, which indicated CCND1 may be a transcriptional target of CCND1 (Figure 6D). Furthermore, miR-145-5p mimics simultaneously inhibited SMAD5 as well as cyclin D1 protein expression (Figure 6E), thus suggesting that miR-145-5p may control SMAD5 expression by affecting cyclin D1. In 41 pairs of GC samples and nearby nontumor tissues, we also found that cyclin D1 expression was present. Additionally, we discovered that GC samples’ expression of CCND1 was substantially lower than that of nearby nontumour tissues (Figures 6F, G). There was a significant positive correlation with SMAD5 expression (Figure 6H). We overexpressed CCND1 in SMAD5-knockdown GC cells to examine whether CCND1 could rescue the impact of low SMAD5 expression on cells. Flow cytometry revealed that CCND1 upregulation significantly reversed the G1 phase arrest caused by SMAD5 knockdown (Figures 6I, J). These findings suggest that SMAD5 potentially regulates the cell cycle by binding to the CCND1 promoter.

## 4 Discussion

Previous studies have explored the function of miR-145-5p in various cancer types and signalling pathways (Lei et al., 2017; Liu et al., 1996; Wei et al., 2017; Jing et al., 2018; Tüzün et al., 2012); however, its role in the proliferation and cell cycle of gastric cancer cells has not been fully characterized. We therefore sought to investigate further the link between miR-145-5p and the cell cycle and GC proliferation. By targeting SMAD5/cyclin D1, miR-145-5p controls the cell cycle and promotes GC development. Ours is the first paper to publish this effect.

GC tissues and cell lines had comparatively lower miR-145-5p expression than normal gastric epithelial cells and tissues, according to our analysis of public databases and miRNA microarrays. Further, there was a positive link of low miR-145-5p expression with advanced TNM staging, high CEA levels, low GC differentiation, and adverse OS rates. These results raise the possibility that miR-145-5p is a GC tumour suppressor gene. These findings are consistent with previous studies showing that lower levels of miR-145-5p expression are associated with poorer patient outcomes and treatment responses (Lei et al., 2017). The forced miR-145-5p expression dramatically lessened GC cell growth in our gain-of-function tests. G1 phase arrest was caused by the miR-145-5p overexpression, which reduced the proportion of S-phase GC cells, according to additional mechanistic research. Nevertheless, additional assessment of the underlying molecular mechanism is required.

Four databases were cross-referenced in order to identify possible gene targets for additional studies. SMAD5 was determined to be the most promising candidate gene. SMAD5 is a member of the receptor-activated SMAD family. As a transcription factor that affects TGF-β signalling, SMAD5 is involved in a variety of physiological and pathological processes, including osteogenic differentiation, proliferation, and metastasis (Liu et al., 1996). For example, Wei et al. reported that SMAD5 regulates the osteogenic differentiation of periodontal ligament stem cells by regulating miR-21 (Wei et al., 2017; Jing et al., 2018; Tüzün et al., 2012). Smad5 inhibits the growth and metastasis of nasopharyngeal carcinoma by binding to miR-384 and inhibiting the Wnt/β-catenin axis (Zeng et al., 2021). SMAD5 also participates in the development of morphine tolerance in bone cancer pain mouse models by regulating miR-93-5p (Xiao et al., 2016). For the first time, we showed that SMAD5 mediates miR-145-5p’s carcinogenic activity in GC by controlling it. We showed by using a dual fluorescein reporter enzyme assay that miR-145-5p binds to SMAD5 directly by attaching to its 3′-UTR. Significantly less SMAD5 protein was expressed when miR-145-5p was overexpressed. In addition, the expression of SMAD5 has been shown to be positively correlated with the tumour grade of gastric cancer (Li et al., 2021). Human GC tissues have higher SMAD5 levels than nearby noncancer tissues. SMAD5 expression and miR-145-5p were negatively connected. GC patients with high SMAD5 expression showed a lower survival rate. These results raise the possibility that SMAD5 is an oncogene in GC. In addition, SMAD5 downregulation dramatically reduced GC cell proliferation, arresting the cell cycle at the G1/S phase. When combined with SMAD5 siRNA, the miR-145-5p inhibitor, however, partially undid the suppression of SMAD5 downregulation. These results imply that by controlling SMAD5, miR-145-5p may prevent the cell cycle and inhibit GC cell proliferation. It is possible that SMAD5 functions as an oncogene in GC. Ours is the first paper that we are aware of that clarifies SMAD5’s role in the cell cycle and GC cell proliferation.

It is well known that cell cycle division in eukaryotes is regulated by cyclins and their catalytic partners (Hydbring et al., 2016). In conjunction with earlier functional experiments, we concentrated on cell cycle-related genes to further validate how SMAD5 affects GC occurrence and development. The protein Cyclin D1, which is encoded by the CCND1 gene, is a central component of the G1 phase that drives cell cycle progression (Ramos-Gar et al., 2017). CCND1 is believed to be an oncogene that accelerates G1/S transformation, thus leading to uncontrolled cell proliferation and tumour progression (Marcolino et al., 2020). High expression of CCND1 is important in tumours. It has been reported that CCND1 amplification and high CCND1 expression can be independent risk factors for liver cancer (Li et al., 2017). Petra et al. reported that CCND1 is involved in the occurrence and proliferation of gastric cancer, and the gene amplification of CCND1 is a potential candidate prognostic marker for the stratification of gastric cancer patients based on survival estimation (Minarikova et al., 2016). The function of SMAD5 in controlling the cell cycle is mediated by cyclin D1, which we discovered to be a transcription target gene of theirs.

Although this study provides important mechanistic insights into the miR-145-5p/SMAD5 axis in GC, certain limitations should be acknowledged. First, the *in vivo* validation remains limited and should be expanded to diverse animal models and larger clinical cohorts. Second, SMAD5 may participate in gastric tumorigenesis through alternative signaling cascades, including but not limited to TGF-β and BMP pathways. Elucidating these potential mechanisms will be critical for a more comprehensive understanding of SMAD5’s role in GC pathophysiology.

## 5 Conclusion

**FIGURE 7:**
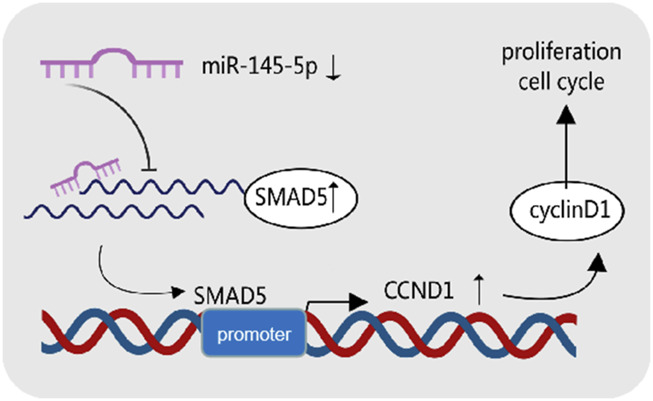
Graphical summary of the mechanism of miR-145-5p in cancer inhibition in gastric cancer cells.

In conclusion, we discovered miR-145-5p′s tumour suppressive function in GC, acting by blocking cell cycle entry and preventing GC cell growth by targeting SMAD5. miR-145-5p′s potential as a gastric cancer biomarker is confirmed by its strong link to a poor GC prognosis. According to our data, GC progression is regulated by the miR-145-5p/SMAD5/CCND1 axis (Figure 7). The molecular mechanisms behind GC progression are better understood thanks to our findings.

## Data Availability

The original contributions presented in the study are included in the article/Supplementary Material, further inquiries can be directed to the corresponding authors.

## Abbreviations

3′UTR, 3′-untranslated region; GC, Gastric cancer; miRNA, microRNA; pri-miRNA, primary miRNA; RNA, ribonucleic acid; IHC, immunohistochemistry; siRNA, short interfering RNA; ceRNAs, competitive endogenous RNAs; qRT-PCR, quantitative reverse transcription polymerase chain reaction.
