# Supplementary material for: MiR-145-5p arrests the cell cycle by modulating SMAD5/cyclin D1 to inhibit gastric cancer progression
Source: Front Cell Dev Biol. 2025 Aug 7;13:1619359. doi: 10.3389/fcell.2025.1619359 (PMC12368585; doi:10.3389/fcell.2025.1619359)
Supplement: Supplementary file 1 [file Table1.docx]

**Table S1 Constructed Sequences Used in This Study**

| miR-145-5p | GUCCAGUUUUCCCAGGAAUCCCU |
| --- | --- |
| miR-145-5p mimic | GUCCAGUUUUCCCAGGAAUCCCU |
|  | GGAUUCCUGGGAAAACUGGACUU |
| miR-145-5p inhibitor | AGGGAUUCCUGGGAAAACUGGAC |
| si-SMAD5-1 | CCAGCAGUAAAGCGAUUGUTT |
|  | ACAAUCGCUUUACUGCUGGTT |
| si-SMAD5-2 | CCACCACUGUCUGUAAGAUTT |
|  | AUCUUACAGACAGUGGUGGTT |
| CCND1 | ATGGAACACCAGCTCCTGTGCTGCGAAGTGGAAACCATCCGCCGCGCGTACCCCGATGCCAACCTCCTCAACGACCGGGTGCTGCGGGCCATGCTGAAGGCGGAGGAGACCTGCGCGCCCTCGGTGTCCTACTTCAAATGTGTGCAGAAGGAGGTCCTGCCGTCCATGCGGAAGATCGTCGCCACCTGGATGCTGGAGGTCTGCGAGGAACAGAAGTGCGAGGAGGAGGTCTTCCCGCTGGCCATGAACTACCTGGACCGCTTCCTGTCGCTGGAGCCCGTGAAAAAGAGCCGCCTGCAGCTGCTGGGGGCCACTTGCATGTTCGTGGCCTCTAAGATGAAGGAGACCATCCCCCTGACGGCCGAGAAGCTGTGCATCTACACCGACAACTCCATCCGGCCCGAGGAGCTGCTGCAAATGGAGCTGCTCCTGGTGAACAAGCTCAAGTGGAACCTGGCCGCAATGACCCCGCACGATTTCATTGAACACTTCCTCTCCAAAATGCCAGAGGCGGAGGAGAACAAACAGATCATCCGCAAACACGCGCAGACCTTCGTTGCCCTCTGTGCCACAGATGTGAAGTTCATTTCCAATCCGCCCTCCATGGTGGCAGCGGGGAGCGTGGTGGCCGCAGTGCAAGGCCTGAACCTGAGGAGCCCCAACAACTTCCTGTCCTACTACCGCCTCACACGCTTCCTCTCCAGAGTGATCAAGTGTGACCCGGACTGCCTCCGGGCCTGCCAGGAGCAGATCGAAGCCCTGCTGGAGTCAAGCCTGCGCCAGGCCCAGCAGAACATGGACCCCAAGGCCGCCGAGGAGGAGGAAGAGGAGGAGGAGGAGGTGGACCTGGCTTGCACACCCACCGACGTGCGGGACGTGGACATC |
