# Supplementary material for: MiR-145-5p arrests the cell cycle by modulating SMAD5/cyclin D1 to inhibit gastric cancer progression
Source: Front Cell Dev Biol. 2025 Aug 7;13:1619359. doi: 10.3389/fcell.2025.1619359 (PMC12368585; doi:10.3389/fcell.2025.1619359)
Supplement: Supplementary file 2 [file Table2.docx]

**Table S2 Primers Used for Quantitative RT-PCR Assays**

| miR-145-5p | Forward | GTCCAGTTTTCCCAGGAATCCCT |
| --- | --- | --- |
|  | Reverse | CTCAACTGGTGTCGTGGAGT |
| SMAD5 | Forward | GAACCCTAAGCTCTGGGAACT |
|  | Reverse | GTGCAAGTCCTCGACCATCC |
| GAPDH | Forward | GTCAAGGCTGAGAACGGGAA |
|  | Reverse | AAATGAGCCCCAGCCTTCTC |
| U6 | Forward | CTCGCTTCGGCAGCACA |
|  | Reverse | AACGCTTCACGAATTTGCGT |
| β-actin | Forward | TGCTGTCCCTGTATGCCTCT |
|  | Reverse | TTGATGTCACGCACGATTTC |
